# Better efficacy of triple antibiotics therapy for human brucellosis: A systematic review and meta-analysis

**DOI:** 10.1371/journal.pntd.0011590

**Published:** 2023-09-14

**Authors:** Shanjun Huang, Hao Wang, Fande Li, Lanping Du, Wenqi Fan, Meifang Zhao, Hua Zhen, Yuke Yan, Menghan Lu, Xin Han, Zhuo Li, Mujinyan Li, Shuqi An, Xinyao Zhang, Qing Zhen, Tiejun Shui

**Affiliations:** 1 Department of Epidemiology and Biostatistics, Key Laboratory of Zoonosis, Ministry of Education, School of Public Health, Jilin University, Changchun, Jilin, PR China; 2 State Key Laboratory for Diagnosis and Treatment of Severe Zoonotic Infectious Diseases, Changchun, China; 3 Yunnan Center for Disease Control and Prevention, Kunming, Yunnan, China; University of Connecticut, UNITED STATES

## Abstract

**Background:**

The treatment of brucellosis suffers from a high recurrence rate and drug resistance. Our study researched the differences in efficacy and side effects between triple antibiotics therapy and dual antibiotics therapy in the treatment of brucellosis through a systematic review and meta-analysis.

**Methods:**

We searched 4 English electronic databases and 2 Chinese electronic databases for randomized controlled trials and cohort studies published through September 2022 on the use of triple antibiotics versus dual antibiotics in the treatment of brucellosis. Overall outcome indicators were therapeutic failure rate, relapse rate, overall therapeutic failure rate, and side effect rate. Relative risk (RR) and 95% confidence intervals (95% CIs) were used as summary statistics. A fixed-effects model was used to combine the overall effect sizes.

**Results:**

The meta-analysis included 15 studies consisting of 11 randomized controlled trials and 4 cohort studies. Triple antibiotics showed better efficacy than dual antibiotics in a comparison of 3 overall outcome indicators (therapeutic failure rate (RR 0.42; 95% CI 0.30 to 0.59 heterogeneity *P* = 0.29, I^2^ = 15%), relapse rate (RR 0.29; 95% CI 0.18 to 0.45 heterogeneity *P* = 0.88, I^2^ = 0%), and overall therapeutic failure rate (RR 0.37; 95% CI 0.28 to 0.48 heterogeneity *P* = 0.35, I^2^ = 9%)). The incidence of side effects in patients with brucellosis treated with triple antibiotics was not significantly different from that in brucellosis patients treated with dual antibiotics (RR 0.85; 95% CI 0.67 to 1.06 heterogeneity *P* = 0.1, I^2^ = 35%). Sensitivity analyses showed robust results and Peter’s test showed no publication bias. The results of subgroup analyses for the research type, drugs, and type of brucellosis were largely consistent with the overall outcome indicators, indicating the reliability and robustness of the overall results.

**Conclusions:**

In the treatment of brucellosis, triple antibiotics have better efficacy than dual antibiotics and do not increase the incidence of side effects.

## Author summary

Brucellosis is a major public health problem in the world. Although rarely fatal, the disease has a tendency to be chronic and persistent, becoming a granulomatous disease capable of affecting any organ system. Therefore, in addition to other control interventions, it is particularly important to provide timely and effective treatment after its occurrence. The current treatment for brucellosis is the dual antibiotics regime recommended by the WHO in 1986 (doxycycline combined with streptomycin or rifampicin). However, classical therapy has a high recurrence rate and increasing rates of drug resistance. In recent years, studies combined classical therapy with another aminoglycoside or quinolone that is triple antibiotic therapy to treat brucellosis, which achieved significant efficacy and no remarkable difference in the incidence of side effects compared to dual antibiotic therapy. Conversely, studies have also found that triple therapy has the same efficacy as dual therapy. In the above context, our study compared the efficacy and safety between the 2 therapies in the treatment of brucellosis, founding that triple antibiotics had better efficacy compared to dual antibiotics and there was no increase in the rate of side effects; both subgroup and sensitive analyses showed that the results were reliable and robust; Peter’s test showed no publication bias in studies. Finally, our study aims to provide an evidence-based basis for treatment options for brucellosis.

## Introduction

Brucellosis is a zoonotic infection caused by the gram-negative, intracellular parasitic bacterium, and is listed by the World Health Organization as one of the “seven neglected endemic zoonotic diseases” [1]. It is estimated that more than 170 countries or regions around the world have brucellosis epidemics, and the number of new cases worldwide is nearly 500,000 or more each year, which not only affects local economic development but also seriously threatens people’s lives and health [2,3]. At the same time, brucellosis in China has a remarkable positive growth rate in 2020 and 2021 in the context of the global pandemic of COVID-19 epidemic in recent years [4]. With such an epidemic trend, we need to pay attention to the prevention, control, and treatment of brucellosis.

Brucellosis has a high rate of initial treatment failure and recurrence, with a large sample study by Bosilkovski and colleagues showing rates of treatment failure and recurrence as high as 10.4% and 16.2% [5]. Although rarely fatal, the ability of the disease to cause cardiac, joint, spinal, and neurological complications have led to a tendency for the disease to be chronic and persistent, becoming a granulomatous disease that can affect any organ system [6–8]. Therefore, the main goals in the treatment of brucellosis are to reduce and shorten the symptomatic period and to prevent or reduce complications, relapse, and chronicity [9,10]. From the initial monotherapy to the 21-day therapy with tetracycline combined with streptomycin recommended by WHO in 1971 [11], the treatment of brucellosis has not achieved satisfactory results because it has a high recurrence rate despite the relief of early symptoms [12,13]. Later in 1986, the WHO established a new brucellosis therapy with doxycycline combined with rifampicin or streptomycin as the first-line regimen [14], and it is still used today. However, relapse still occurs in up to 10% of patients treated with the recommended drugs [15,16], and it has also raised concerns about the increased drug resistance due to the long-term use of rifampin that is often used as one of the drugs for tuberculosis treatment [17]. A series of problems have led to calls for new drug treatment options.

Research has shown increasing rates of recurrence and drug resistance in brucellosis [18]. Rifampicin-resistant strains have been detected to varying degrees in China, Norway, and Turkey using E-Test [19]. In Egypt, patients with osteoarthritic brucellosis treated with rifampicin in combination with doxycycline for 5 months had a recurrence rate of 59.3% [20]. Therefore, there have been attempts by specialists to use a triple antibiotic regimen. For acute brucellosis, a combination of doxycycline, rifampicin, and streptomycin can achieve a 100% cure rate [21]; no cases of relapse after combined treatment with doxycycline and rifampin plus aminoglycosides for osteoarthritic brucellosis [22]; long-term triple therapy is also effective in patients with spinal bone destruction caused by brucellosis [23]. However, it has also been found that triple antibiotic therapy has the same clinical effect as dual antibiotic therapy in the treatment of brucellosis [24,25]; despite the higher efficacy of triple antibiotic therapy, the former has a higher rate of side effects compared to the dual antibiotic therapy regimen [15].

Studies have quantified and compared the effect of dual antibiotic combination regimens on the treatment of brucellosis [10,26]. Regarding triple antibiotics versus dual antibiotics, 2 studies and 1 study were included in the above systematic review and meta-analysis, respectively. Limited by the number of studies, it remains to be seen whether triple antibiotic therapy is superior to dual antibiotic therapy in the treatment of brucellosis. Therefore, this study specifically quantified the effectiveness of triple antibiotics compared with dual antibiotics for the treatment of brucellosis, hoping to provide an evidence-based basis for the clinical treatment of brucellosis.

## Methods

### Protocol and registration

The results of the systematic review and meta-analysis followed the Preferred Reporting Items for Systematic Reviews and Meta-Analyses (PRISMA) 2020 statement [27]. The study protocol was registered in PROSPERO (CRD42022362810). See **Texts S1 and S2** for the PRISMA Checklist.

### Search strategy

We searched all English and Chinese literature for randomized controlled trials and cohort studies published through September 2022 on the use of triple antibiotics versus dual antibiotics in the treatment of patients with brucellosis. Four English databases (PubMed, Web of Science, CENTRAL, and Embase) and 2 Chinese databases (China National Knowledge Infrastructure (CNKI) and SinoMed) were searched. Searched characters using the following keywords: “Brucellosis,” “Brucella,” “Malta Fever,” “Gibraltar Fever,” “Therapy,” “Treatment,” etc. Based on different databases, the search strategies were adjusted accordingly by multiple tests combining the MeSH and free words. The search strategies used for English databases are shown in the **S3 Text**. We also reviewed the references in the included studies to ensure the inclusion of all relevant literature.

### Outcome indicators

Our 2 predefined primary outcomes were “relapse,” defined as the reappearance of relevant clinical symptoms, rise in antibody titers or positive results on cultures after the end of treatment, during the follow-up period; and “overall failure,” defined as the sum of relapse and therapeutic failure. Secondary outcome indicators include therapeutic failure and side effects. Therapeutic failure is defined as a patient’s symptoms not improving by the end of treatment. Side effects are defined as uncomfortable symptoms that occur during the administration of the drug, such as nausea and vomiting, abdominal pain and diarrhea, etc., serious side effects such as ototoxicity, hepatotoxicity, nephrotoxicity, and skin reactions.

### Inclusion and exclusion criteria

Articles were included in the meta-analysis if they met the following criteria: (1) a study of triple antibiotics therapy for brucellosis; and (2) a randomized controlled trial or cohort study of drug therapy for brucellosis. Those articles were excluded if they met: (1) The control group in the study was not dual antibiotics; (2) the study population includes pregnant women or children; and (3) a combination of serious complications, such as endocarditis or neurological disease (excluding osteoarticular brucellosis). To ensure an adequate sample, studies that lacked a particular primary outcome indicator or secondary outcome indicator are not used as exclusion criteria but were simply not included in the subgroup analysis.

### Data extraction and quality assessment

The selection of the literature, extraction of data, and assessment of the quality of the literature was strictly performed by 2 reviewers in accordance with the inclusion and exclusion criteria and the Cochrane Handbook. Differences in interpretation by the 2 reviewers were resolved by discussion and consensus. The extracted data include basic information about the study (authors, publication date, region, inclusion and exclusion criteria of patients, etc.), information on quality assessment (random method, allocation concealment, blinding of participants and personnel, blinding of outcome assessment, completeness of result data, selective reporting, etc.), and original data (therapy, number of patients, relapses, treatment failures, side effects, and loss of follow-up, etc.). Risk of bias assessment was undertaken by us using RevMan5.4 software, according to the RCT bias risk assessment tool recommended by the Cochrane Collaboration Handbook. In addition, we used the Newcastle–Ottawa scale (NOS) [28] to assess the quality of the included cohort studies.

### Statistical analysis

We used R version 4.2.1 to merge and analyze the data. Relative risk (RR) and 95% confidence intervals (95% CIs) were used as summary statistics. We used a χ2 test of heterogeneity and the I^2^ measure of inconsistency to assess heterogeneity in the results of the trials. If the heterogeneity of the combined results is significant (χ2 test *P* < 0.1 or I^2^ > 50%), a random effects model will be used to pool the data. Otherwise, the fixed effects model will be used to combine the data. We used visual funnel plots to qualitatively observe publication bias. Given the low heterogeneity of our study, the number of included studies exceeding 10, and the qualitative data, Peter’s test was chosen for quantitative judgment [29].

### Subgroup analysis

Regardless of the heterogeneity of the results, we will perform subgroup and sensitivity analyses to assess the effect of subgroup factors on the results and the stability of the results. Our predetermined subgroup comparisons were drugs (doxycycline combined with rifampin plus any quinolone versus doxycycline plus rifampin, doxycycline combined with rifampin plus any aminoglycoside versus doxycycline plus rifampin), research type (RCT versus cohort study), and brucellosis type (uncomplicated brucellosis versus osteoarticular brucellosis).

## Results

### Search result

**Fig 1:**
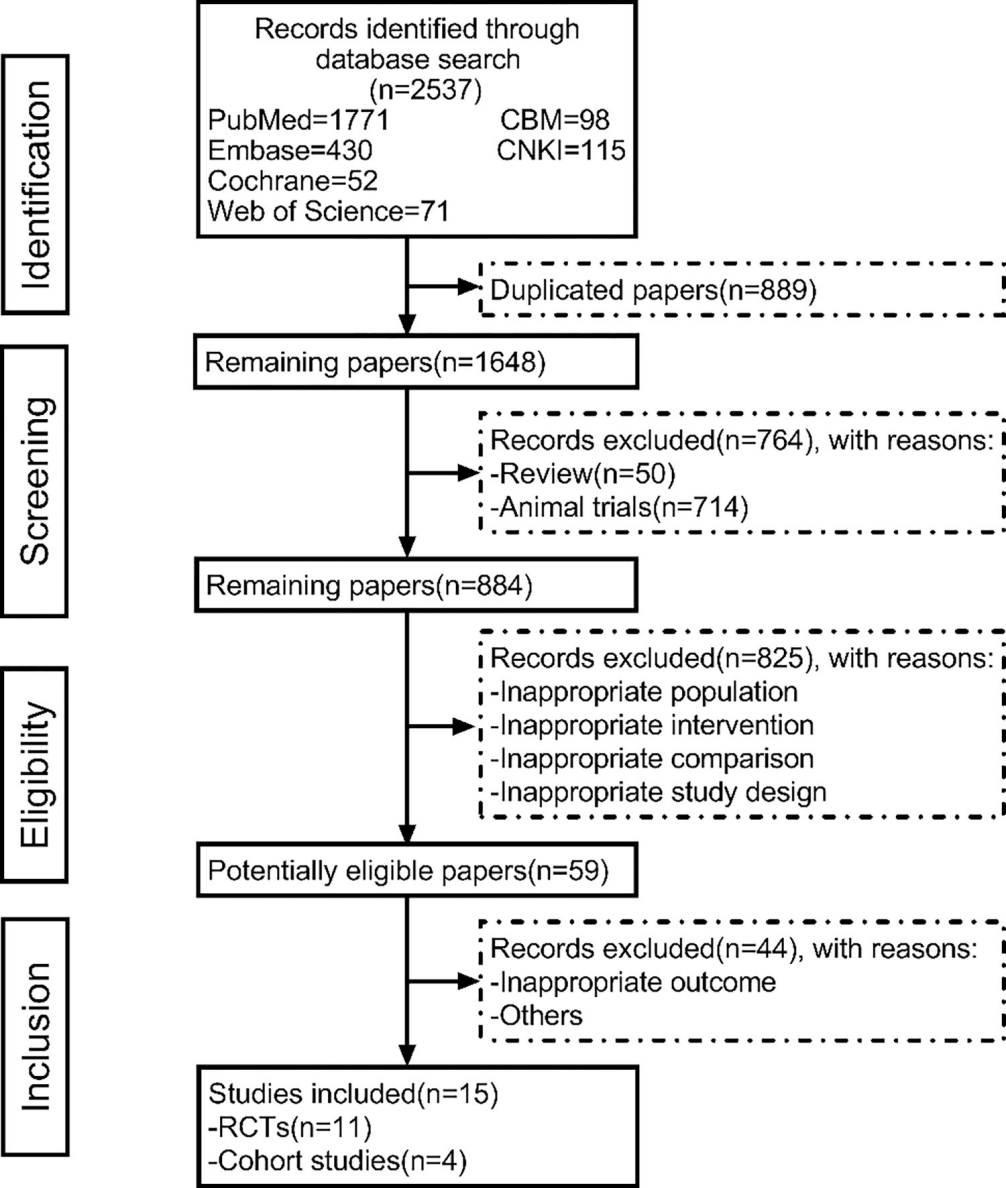
Flowchart of study selection.

A total of 2,537 papers were searched according to the search strategy. After removing duplicates and following the inclusion and exclusion criteria, we finally included 15 papers that were obtained from PubMed, Web of Science, CENTRAL, Embase, CNKI, and SinoMed databases in the meta-analysis (total of 1,466 patients and 35 treatment groups), consisting of 11 randomized controlled trials and 4 cohort studies [15,21,22,24,25,30–39]. **Fig 1** shows the flow chart of the literature screening process.

### Characteristics of included studies

**Fig 2:**
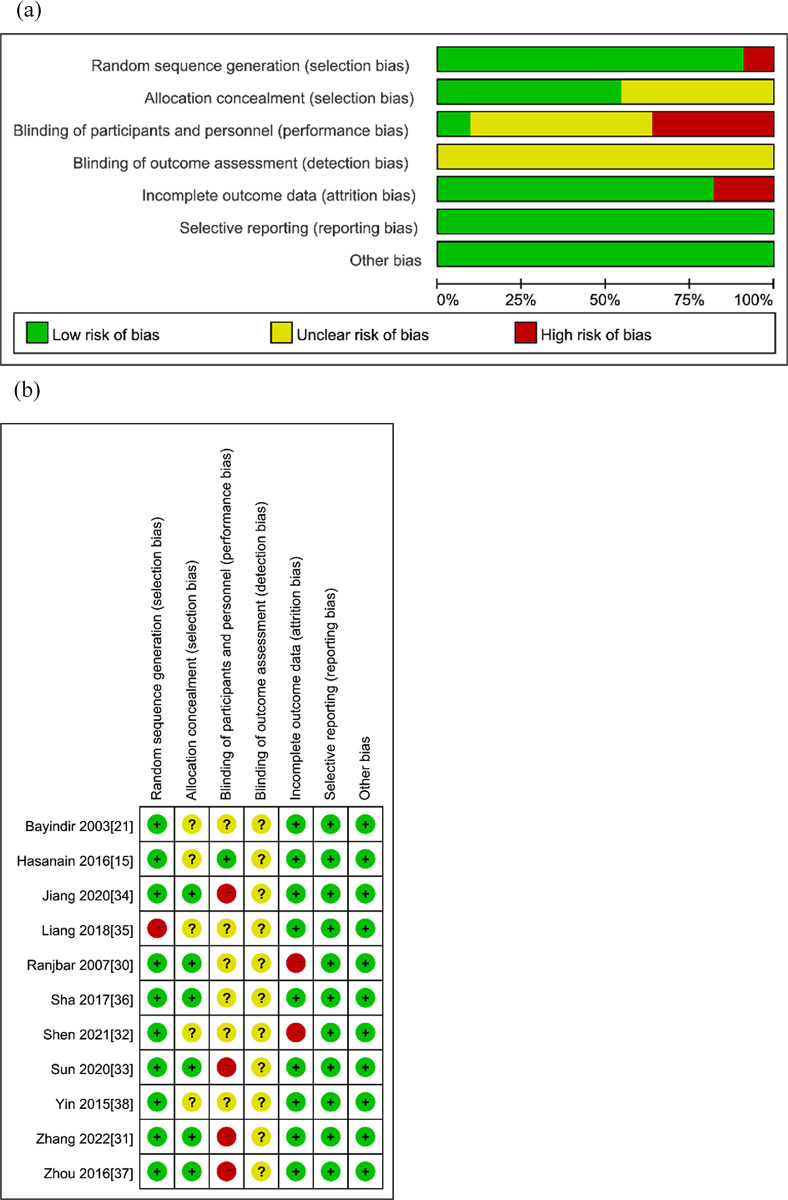
Assessment quality of RCTs. (a) Risk of bias graph. (b) Risk of bias summary.

Four of the included publications had osteoarticular brucellosis, the rest were patients with acute or subacute uncomplicated brucellosis and did not include children or pregnant women. **Tables 1 and S1** demonstrate the basic characteristics of the included literature. In terms of reporting of outcome indicators, 14 literature reported therapeutic failure rate, 11 literature reported relapse rate, 15 literature reported overall therapeutic failure rate, and 13 literature reported side effect rate. In the 11 randomized controlled trials, 6 reported allocation concealment and no literature reported blinding method (**Fig 2**). Since the studies were all three-drug combinations compared to two-drug combinations, and the outcome indicators were objective except for side effects, it can be assumed that bias caused by allocation concealment and improper implementation of blinding had little impact on the experimental results. The specific items and the assessment results of cohort study are presented in **Table 2.**

**Table 1:** Characteristics of included studies.

Trial	Interventions	Area	Patients	Mean age	Man%	Disease type	Side effects	Research type
Hasanain 2016 [15]	D 200 mg for 6 weeks+R 900 mg for 6 weeks+L 500 mg for 6 weeks vs. D 200 mg for 6 weeks+R 900 mg for 6 weeks	Assiut	107	34.5±16.3	57.9	Uncomplicated	epigastric pain; nausea and vomiting; diarrhea; fatigue; rash; heartburn; oral candidiasis	RCT
Ranjbar 2007 [30]	D 2 × 100 mg for 8 weeks+R 10 mg/kg/day for 8 weeks+A 2 × 7.5 mg/kg/day for 7 days vs. D 2 × 100 mg for 8 weeks+R 10 mg/kg/day for 8 weeks	Iran	220	35.7±17	48.6	Uncomplicated	vomiting; phototoxicity; gastric complaints; genital candidiasis	RCT
Bayindir 2003 [21]	D 2 × 100 mg for 45 days+R 15 mg/kg/day for 45 days+S 1 g for 15 days vs. D 2 × 100 mg for 45 days+R 15 mg/kg/day for 45 days	Turkey	42	41.3	54.8	Brucellar spondylitis	gastrointestinal complaints (heartburn, epigastric pain, nausea, and vomiting)	RCT
	D 2 × 100 mg for 45 days+R 15 mg/kg/day for 45 days+S 1 g for 15 days vs. D 2 × 100 mg for 45 days+S 1 g for 15 days		43	40.19	53.5			
	D 2 × 100 mg for 45 days+R 15 mg/kg/day for 45 days+S 1 g for 15 days vs. S 1 g for 15 days+T 4 × 500 mg for 45 days		42	43.13	57.1			
	D 2 × 100 mg for 45 days+R 15 mg/kg/day for 45 days+S 1 g for 15 days vs. L 2 × 200 mg for 45 days+R 15 mg/kg (600–900 mg/d) for 45 days		41	39.62	53.7			
Zhang 2022 [31]	D 2 × 100 mg for 6 weeks+R 900 mg for 6 weeks+L 500 mg for 6 weeks vs. D 2 × 100 mg for 6 weeks+R 900 mg for 6 weeks	China	76	49.47±3.09	51.3	Uncomplicated	gastrointestinal complaints; allergy; liver and kidney injury	RCT
Shen 2021 [32]	D+R+S vs. RT (dose is unavailable)	China	70	37.73±3.32	45.7	Brucellar spondylitis	drug-related hepatitis; gastrointestinal complaints	RCT
Sun 2020 [33]	D 2 × 100 mg for 6 weeks+R 600 mg for 6 weeks+L 500 mg for 6 weeks vs. D 2 × 100 mg for 6 weeks+R 600 mg for 6 weeks	China	60	45.69±3.03	53.3	Uncomplicated	liver function impairment; gastrointestinal complaints; rash; leukopenia	RCT
Jiang 2020 [34]	D 2 × 100 mg for 6 weeks+R 600 mg for 6 weeks+L 2 × 200 mg for 7 days vs. D 2 × 100 mg for 6 weeks+R 600 mg for 6 weeks	China	74	42.25±16.33	56.8	Uncomplicated	rash; liver function impairment; gastrointestinal complaints; leukopenia	RCT
Liang 2018 [35]	D 100 mg for 4 weeks+R 2 × 450 mg for 4 weeks+L 400 mg for 4 weeks vs. D 100 mg for 4 weeks+R 2 × 450 mg for 4 weeks	China	76	30.35±5.34	55.3	Uncomplicated	rash; nausea and vomiting; dizziness	RCT
Sha 2017 [36]	D 100 mg for 6 weeks+R 600 mg for 6 weeks+L 400 mg for 6 weeks vs. D 100 mg for 6 weeks+R 600 mg for 6 weeks	China	112	46±7.12	77.7	Uncomplicated	gastrointestinal complaints; rash; liver function impairment	RCT
Zhou 2016 [37]	D 2 × 100 mg for 6 weeks+R 600 mg for 6 weeks+L 500 mg for 6 weeks vs. D 100 mg for 6 weeks+R 600 mg for 6 weeks	China	120	47.76±11.5	78.3	Uncomplicated	gastrointestinal complaints	RCT
Yin 2015 [38]	D 2 × 100 mg for 6 weeks+R 600 mg for 6 weeks+L 400 mg for 7 days vs. D 100 mg for 6 weeks+R 600 mg for 6 weeks	China	64	42±18.60	68.8	Uncomplicated	gastrointestinal complaints; rash	RCT
Mile 2012 [25]	D 200 mg for 45 days+R 900 mg for 45 days+G 2 × 120 mg for 7–10 days vs. D 200 mg for 45 days+R 900 mg for 45 days (100 mg, 600 mg, 160 mg for those with a body weight of less than 40 kg)	Macedonia	181	33	71.2	Uncomplicated	gastrointestinal complaints; generalized hypersensitivity reactions; gastrointestinal disturbances	Cohort study
Al-Madfaa 2020 [24]	D 2 × 100 mg for 45 days+R 3 × 300 mg or 1 × 900 mg for 45 days+S 1,000 mg for 7 days vs. D 2 × 100 mg for 45 days+R 3 × 300 mg or 1 × 900 mg for 45 days	Saudi Arabia	54	49.2±19.4	53.7	Uncomplicated	gastric discomfort; esophagitis; heartburn; severe pain; gastrointestinal upset	Cohort study
Yang 2021 [39]	D 100 mg for 4 weeks+R 450 mg for 4 weeks+C 100 mg for 4 weeks vs. R 450 mg for 4 weeks+C 100 mg for 4 weeks	China	100	43.3±5.1	54	Brucellar spondylitis	gastrointestinal complaints; rash; liver damage; kidney function damage	Cohort study
Smailnejad 2012 [22]	D 2 × 100 mg+R 15 mg/kg/day+C 8 mg/kg/day vs. D 2 × 100 mg+R 15 mg/kg/day	Iran	31	NA	NA	Brucellar spondylitis	no serious adverse effects were observed during treatment	Cohort study
	D 2 × 100 mg+R 15 mg/kg/day+G 5 mg/kg/day (up to 240 mg) vs. D 2 × 100 mg+R 15 mg/kg/day		26					
	D 2 × 100 mg+R 15 mg/kg/day+S 1,000 mg/day vs. D 2 × 100 mg+R 15 mg/kg/day		33					

C, co-trimoxazole; D, doxycycline; G, gentamicin; L, levofloxacin; NA, not available; R, rifampicin; S, streptomycin; T, tetracycline-HCl.

**Table 2:** Assessment of quality of included cohort studies (NOS).

Study	Selection				Comparability	Exposure			Scores
	Representativeness of the exposed cohort	Selection of the non-exposed cohort	Ascertainment of exposure	Demonstration that outcome of interest was not present at start of study	Comparability of cohorts on the basis of the design or analysis	Assessment of outcome	Was follow-up long enough for outcomes to occur	Adequacy of follow-up of cohorts	
Mile 2012 [25]	0	1	1	1	0	0	1	1	5
Al-Madfaa 2020 [24]	0	1	1	0	2	0	0	0	4
Yang 2021 [39]	0	1	1	0	2	0	0	0	4
Smailnejad 2012 [22]	1	1	1	1	0	0	1	1	6

### Comparison of overall outcome indicators

**Fig 3:**
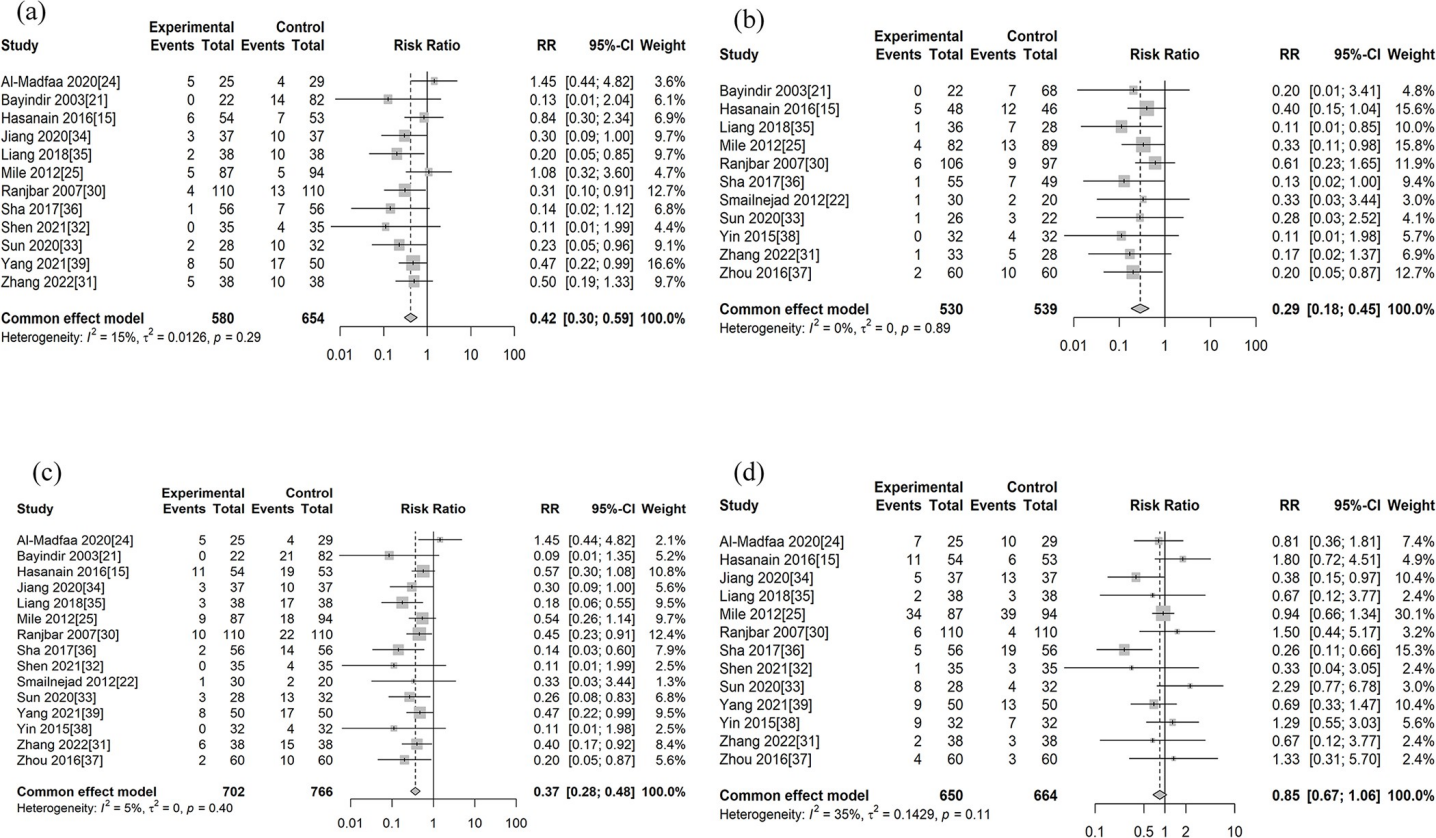
Forest plot of overall outcome indicators. (a) Therapeutic failure rate. (b) Relapse rate. (c) Overall therapeutic failure. (d) Side effect rate.

Therapeutic failure rate: 12 literature reported therapeutic failure rate (the therapeutic failure rate for both the treatment and control groups in the 2 papers was 0%; therefore, it is not included in the analysis), which included a total of 1,234 patients. The result showed that the therapeutic failure rate for triple antibiotics was lower than that for dual antibiotics (RR 0.42; 95% CI 0.30 to 0.59), with mild heterogeneity (*P* = 0.29, I^2^ = 15%) (**Fig 3A**).

Relapse rate: 11 literature reported relapse rate, which included a total of 1,069 patients. Triple antibiotics regimen resulted in a significantly lower rate of relapse than the dual antibiotics regimen (RR 0.29; 95% CI 0.18 to 0.45), with no heterogeneity (*P* = 0.88, I^2^ = 0%) (**Fig 3B**).

Overall therapeutic failure rate: 15 literature reported this indicator, which included a total of 1,468 patients. The result showed that the overall therapeutic failure rate for triple antibiotics was lower than that for dual antibiotics (RR 0.37; 95% CI 0.28 to 0.48), with mild heterogeneity (*P* = 0.35, I^2^ = 9%) (**Fig 3C**).

Side effect rate: 13 literature reported this indicator, which included a total of 1,314 patients. The results showed no statistically significant difference in the rate of side effects between triple antibiotics therapy and dual antibiotics therapy (RR 0.85; 95% CI 0.67 to 1.06), with some heterogeneity (*P* = 0.1, I^2^ = 35%) (**Fig 3D**).

Sensitivity analyses were conducted for the studies included in each of the above overall outcome indicators, suggesting robust results (**S1 Fig**). What’s more, S2 **Fig** shows the results of the Funnel Chart. After Peter’s test, the results showed that all *P* values were greater than 0.05 (therapeutic failure rate: *P* = 0.9999, relapse rate: *P* = 0.0892, overall therapeutic failure rate: *P* = 0.6943, side effect rate: *P* = 0.9401).

### Results of subgroup analysis

Subgroup analysis further explored whether subgroups (research type, drugs, brucellosis type) had an effect on the overall outcome indicators (**Table 3**).

**Table 3:** Results of subgroup analysis of triple antibiotics compared to dual antibiotics.

Subgroups	No.of studies (cases	Rate of therapeutic failure			No. of studies (cases)	Rate of relapse			No. of studies (cases)	Rate of total therapeutic failure			No. of studies (cases)	Rate of adverse reaction		
		RR (95% CI)	I^2^	*P*		RR (95% CI)	I^2^	*P*		RR (95% CI)	I^2^	*P*		RR (95% CI)	I^2^	*P*
Research type																
RCT	9 (899)	0.32 (0.20–0.49)	0%	0.59	9 (848)	0.27 (0.17–0.46)	0%	0.74	11 (1,083)	0.30 (0.22–0.42)	0%	0.49	10 (979)	0.82 (0.59–1.15)	50%	0.04
Cohort study	3 (335)	0.73 (0.42–1.25)	33%	0.22	2 (221)	0.33 (0.13–0.89)	0%	1	4 (385)	0.58 (0.37–0.93)	0%	0.43	3 (335)	0.87 (0.64–1.17)	0%	0.75
Regimen																
DR+Q vs. DR	6 (505)	0.36 (0.22–0.58)	0%	0.42	7 (555)	0.22 (0.12–0.40)	0%	0.86	8 (689)	0.30 (0.21–0.44)	0%	0.43	8 (689)	0.80 (0.56–1.14)	57%	0.02
DR+A vs. DR	4 (497)	0.60 (0.32–1.10)	45%	0.14	5 (472)	0.42 (0.22–0.81)	0%	0.87	6 (556)	0.51 (0.33–0.79)	0%	0.45	3 (455)	0.96 (0.70–1.32)	0%	0.71
Disease type																
Uncomplicated	9 (960)	0.45 (0.31–0.66)	29%	0.18	9 (929)	0.29 (0.18–0.46)	0%	0.75	11 (1,144)	0.38 (0.29–0.51)	22%	0.23	11 (1,144)	0.88 (0.69–1.11)	42%	0.07
Osteoarticular	3 (274)	0.33 (0.16–0.68)	0%	0.39	2 (140)	0.25 (0.04–1.61)	0%	0.78	4 (324)	0.30 (0.15–0.61)	0%	0.45	2 (170)	0.62 (0.31–1.27)	0%	0.54

DR+Q (doxycycline plus rifampin in combination with quinolones); DR+A (doxycycline plus rifampin in combination with aminoglycosides).

Research type: Performing subgroup analysis by study type, we found that both RCT and cohort studies presented the same results as the overall outcome indicators results in terms of relapse rate, overall therapeutic failure rate, and side effect rate. In other words, the relapse rate (RR 0.27, 95% CI 0.17 to 0.46; RR 0.33, 95% CI 0.13 to 0.89) and the overall therapeutic failure rate (RR 0.30, 95% CI 0.22 to 0.42; RR 0.58, 95% CI 0.37 to 0.93) were lower for triple antibiotics than for dual antibiotics. The difference in side effect rates was not statistically significant (RR 0.82, 95% CI 0.59 to 1.15; RR 0.87, 95% CI 0.64 to 1.17). For treatment failure rate, the RCT group showed the same result as the overall combined effect size (RR 0.32, 95% CI 0.20 to 0.49), in contrast to the results shown in the cohort studies (RR 0.73, 95% CI 0.42 to 1.25).

Drugs: The results of treatment failure, relapse, and adverse reaction rates by regimen and duration of treatment are summarized in **S2 Table**. A total of 8 of the included papers reported treatment measures of doxycycline plus rifampin in combination with quinolones (DR+Q) versus doxycycline plus rifampin (DR) and 6 reported the effect of doxycycline plus rifampin in combination with aminoglycosides (DR+A) versus doxycycline plus rifampin. Two of the 3 categories of combination drugs reported in Smailnejad 2012 meet the requirements for comparison and are therefore classified as Smailnejad 2012a and Smailnejad 2012b. Subgroup analysis showed that the combination of quinolones or aminoglycosides on top of doxycycline plus rifampicin was more effective than doxycycline plus rifampicin alone, mainly in terms of lower relapse rates (RR 0.22, 95% CI 0.12 to 0.40; RR 0.42, 95% CI 0.22 to 0.81) and overall therapeutic failure rates (RR 0.30, 95% CI 0.21 to 0.44; RR 0.51, 95% CI 0.33 to 0.79), and statistically nonsignificantly different in side effects rate between them (RR 0.80, 95% CI 0.56 to 1.14; RR 0.96, 95% CI 0.70 to 1.32). However, in terms of therapeutic failure rates (RR 0.60, 95% CI 0.32 to 1.10), triple therapy combined with aminoglycosides did not show the expected effect.

Brucellosis type: In included studies, 4 papers were studied in patients with osteoarticular brucellosis. We performed a subgroup analysis to assess the efficacy between triple therapy and dual therapy in patients with osteoarticular brucellosis and uncomplicated brucellosis. The results showed that triple antibiotics therapy had lower therapeutic failure rates (RR 0.45, 95% CI 0.31 to 0.66; RR 0.33, 95% CI 0.16 to 0.68) and overall therapeutic failure rates (RR 0.38, 95% CI 0.29 to 0.51; RR 0.30, 95% CI 0.15 to 0.61) for uncomplicated brucellosis and osteoarticular brucellosis than for dual antibiotics therapy. The differences in the side effects rate exhibited by the 2 therapies in the treatment of uncomplicated brucellosis (RR 0.88, 95% CI 0.69 to 1.11) and osteoarticular brucellosis (RR 0.62, 95% CI 0.31 to 1.27) were not statistically significant. However, in terms of relapse rate in the treatment of osteoarticular brucellosis, the results showed no significant difference between triple therapy and dual therapy (RR 0.25, 95% CI 0.04 to 1.61).

## Discussion

In this study on the treatment of brucellosis, we compared the differences in treatment efficacy and safety between triple antibiotics and dual antibiotics in 15 papers based on 4 overall outcome indicators, in terms of research type, drugs, and brucellosis type. In a comparison of 3 overall outcome indicators (therapeutic failure rate, relapse rate, and overall therapeutic failure rate), triple antibiotics showed better efficacy than dual antibiotics, sensitivity analyses showed robust results and Peter’s test showed no publication bias. The usage of more antibiotics might increase the incidence of side effects; however, in our study, the incidence of side effects in patients with brucellosis treated with triple antibiotics was not significantly different from that in brucellosis patients treated with dual antibiotics, suggesting that triple antibiotics have better therapeutic value compared to the dual antibiotics. In previous systematic reviews and meta-analysis [10,26], triple antibiotics were also suggested to have better efficacy but were limited by low sample size (2 studies and 1 study) and had limited reference value. The present study, however, included a larger number of study subjects and is, therefore, more referable to some extent.

In the subgroup analysis, the results of most subgroups were consistent with the overall results, all favoring triple antibiotic therapy more. However, opposite results were shown for some indicators, which we will explain for these situations. Firstly, in the analysis of the research type subgroup, the cohort study group suggested no significant difference between triple antibiotics and dual antibiotics in terms of therapeutic failure rate, which is the opposite of the results for both the RCT group and overall outcome indicators. In this case, we refer to Park and colleagues [40–42], arguing that RCTs have a higher level of evidence and therefore would be more inclined to the conclusions of the RCT studies. Secondly, in a subgroup analysis of drug therapy, the triple drugs of DR combined with aminoglycosides group were not significantly different from the DR group in terms of therapeutic failure rate. We reviewed the 4 studies included in this subgroup and found a greater proportion of critically ill patients in the triple antibiotics group in the study of Al-madfaa (60% versus 37.9%). Although this difference is not statistically significant, it may mask the potential benefits of triple therapy to some extent. Finally, in a subgroup comparison between triple antibiotics and dual antibiotics for the relapse rate of osteoarticular brucellosis, the results showed no significant difference, which is the opposite of the results shown for the uncomplicated brucellosis treatment group and overall outcome indicators. We found that the analysis of this group included only 2 studies (Bayindir (2003) and Smailnejad (2012)) with a total of 140 patients (52 versus 88), which is too small a sample size for possible false negative results.

This study is the first systematic review and meta-analysis to summarize the differences in efficacy and safety of triple antibiotics therapy versus dual antibiotics therapy for brucellosis, and there are some limitations. For example, the study did not use demographic characteristics as a grouping factor in conducting subgroup analysis (including region, gender, etc.). We were unable to obtain a more precise analysis of the different clinical stages of brucellosis because some of the included literature did not clearly describe the clinical stage and age classification of brucellosis. What’s more, most of the drug combinations included in this study were DRQ (doxycycline, rifampin, quinolones) or DRA (doxycycline, rifampin, aminoglycosides), and the differences between these 2 triple combination regimens and the 2 combination regimens (DR) were compared separately, and it is not known whether other drug combinations such as those containing ceftriaxone have better therapeutic effects. In addition, our study did not specifically analyze the duration of treatment. The choice of antibiotic regimen should be based on the presence of focal disease or the presence of certain contraindications and potential populations for which antibiotics are contraindicated [43]. Therefore, children and pregnant women were excluded from our study, and all patients with brucellosis do not have focal lesions. For children and pregnant women, as well as for brucellosis patients with combined endocarditis and neurological symptoms, refer to the appropriate treatment regimen.

Although this meta-analysis suggests that triple antibiotics therapy is the superior choice compared to the traditional dual antibiotics therapy; however, it is also important to consider drug acceptability and economic factors in the choice of treatment regimen, considering that the majority of brucellosis cases currently occur in lower socioeconomic areas and that patients are mainly farmers, pastoralists, and people who are exposed to infected animal products [44]. For the acceptability of the relevant drug, we found that relatively cheap and easily accessible drug formulations are often chosen as first-line prescriptions in the real world in order to control drug costs [45]. Furthermore, even experts tend to prefer convenience over mere scientific advantage. Meanwhile, this trend is independent of disease experience or country of origin [46]. One study showed that 64.6% of healthcare professionals interviewed preferred the doxycycline-rifampicin regimen for human brucellosis [46], despite the demonstrated superiority of doxycycline-streptomycin [43,47]. The reason for this may be the lower price and ease of administration of rifampicin [7,46]; in contrast, streptomycin requires parenteral administration in a hospital setting or in an appropriately set-up primary care network, both of which are restricted in low-income countries [48]. Also, economic factors can affect patient compliance. We found that out-of-pocket health expenditures are a major source of healthcare financing in low- and middle-income countries (LMICs) and are strongly correlated with antimicrobial resistance in LMICs [49]. Moreover, poverty encourages shorter courses of treatment, sharing of medicines, or the use of lower quality or expired medicines [50]. This inappropriate allocation and timing of antibiotics often lead to treatment failure or resistance [51]. In addition, according to a study by Straight and Martin in 2002 [52] on the cost of drug treatment for brucellosis, quinolones were the most expensive drugs, costing approximately $224.06 for 45 days of treatment. Gentamicin is cheaper than streptomycin, although, for both drugs, the cost of parenteral administration equipment must be increased. Most of the triple antibiotic regimens are combined with aminoglycosides or quinolones on top of classical regimens, so for some poor areas, triple antibiotic regimens may not be the preferred treatment option. Studies undertaken to determine the best form of treatment for these patients should take into account social and economic factors [53], and it is difficult to conduct such studies from the perspective of countries where drug administration, follow-up, or availability of antibiotics pose a problem [26]. This may also be the reason why only 1 continental African region was included in our study.

It has to be mentioned that we did not perform a subgroup analysis of the specific grading of side effects and duration of treatment. Even so, our study is valuable in identifying the superior efficacy of triple antibiotics therapy versus dual antibiotics therapy. In the future, an in-depth study of the duration of triple antibiotic treatment and the grading of side effects will be a very meaningful topic.

In conclusion, the choice of a treatment plan for brucellosis requires consideration of a variety of factors, such as the dose and course of the drugs, the socioeconomic situation, the availability of drugs, and even the traditional treatment protocols of medical institutions and the clinical experience of physicians. Therefore, the choice of treatment options for brucellosis should be diverse. Finally, our study aims to provide evidence-based medicine for the treatment of brucellosis, and more randomized controlled trials of rigorous design, preferably large, international, multicenter clinical studies, are required to be included in the future.

## Supporting information

S1 Text PRISMA Abstract Checklist. (DOCX)

S2 Text PRISMA 2020 Checklist. (DOCX)

S3 Text Search strategy. (DOCX)

S1 Table Characteristics of included studies. (DOCX)

S2 Table Treatment failure, relapse, and adverse reaction rates by regimen and duration of treatment. (DOCX)

S1 Fig Sensitivity analysis (Peter’s test). (DOCX)

S2 Fig Funnel plot. (DOCX)
