# Supplementary material for: Better efficacy of triple antibiotics therapy for human brucellosis: A systematic review and meta-analysis
Source: PLoS Negl Trop Dis. 2023 Sep 14;17(9):e0011590. doi: 10.1371/journal.pntd.0011590 (PMC10501551; doi:10.1371/journal.pntd.0011590)
Supplement: S3 Text — (DOCX) [file pntd.0011590.s003.docx]

**S1. Search strategy.**

**PubMed(20/09/2022)**

| **Search** | **Query** |
| --- | --- |
| #8 | #4 AND #7 |
| #7 | #5 OR #6 |
| #6 | ((((Therapeutic[Title/Abstract]) OR (Therapy[Title/Abstract])) OR (Therapies[Title/Abstract])) OR (Treatment[Title/Abstract])) OR (Treatments[Title/Abstract]) |
| #5 | "Therapeutics"[Mesh] |
| #4 | #1 OR #2 OR #3 |
| #3 | (((((((((((((((((Brucelloses[Title/Abstract]) OR (Malta Fever[Title/Abstract])) OR (Fever, Malta[Title/Abstract])) OR (Gibraltar Fever[Title/Abstract])) OR (Fever, Gibraltar[Title/Abstract])) OR (Rock Fever[Title/Abstract])) OR (Fever, Rock[Title/Abstract])) OR (Cyprus Fever[Title/Abstract])) OR (Fever, Cyprus[Title/Abstract])) OR (Brucella Infection[Title/Abstract])) OR (Brucella Infections[Title/Abstract])) OR (Infection, Brucella[Title/Abstract])) OR (Undulant Fever[Title/Abstract])) OR (Fever, Undulant[Title/Abstract])) OR (Brucellosis, Pulmonary[Title/Abstract])) OR (Brucelloses, Pulmonary[Title/Abstract])) OR (Pulmonary Brucelloses[Title/Abstract])) OR (Pulmonary Brucellosis[Title/Abstract]) |
| #2 | "Brucella"[Mesh] |
| #1 | "Brucellosis"[Mesh] |

**Web of Science(20/09/2022)**

TS=( Brucellosis OR Brucelloses OR Malta Fever OR Fever, Malta OR Gibraltar Fever OR Fever, Gibraltar OR Rock Fever OR Fever, Rock OR Cyprus Fever OR Fever, Cyprus OR Brucella Infection OR Brucella Infections OR Infection, Brucella OR Undulant Fever OR Fever, Undulant OR Brucellosis, Pulmonary OR Brucelloses, Pulmonary OR Pulmonary Brucelloses OR Pulmonary Brucellosis) AND TS=( Therapeutics OR Therapeutic OR Therapy OR Therapies OR Treatment OR Treatments)

**Embase(20/09/2022)**

('Brucellosis'/exp OR 'Brucella'/exp OR Brucelloses:ab,ti OR 'Malta Fever':ab,ti OR 'Malta Fever':ab,ti OR 'Fever, Malta':ab,ti OR 'Gibraltar Fever':ab,ti OR 'Fever, Gibraltar':ab,ti OR 'Rock Fever':ab,ti OR 'Fever, Rock':ab,ti OR 'Cyprus Fever':ab,ti OR 'Fever, Cyprus':ab,ti OR 'Brucella Infection':ab,ti OR 'Brucella Infections':ab,ti OR 'Infection, Brucella':ab,ti OR 'Undulant Fever':ab,ti OR 'Fever, Undulant':ab,ti OR 'Brucellosis, Pulmonary':ab,ti 'Brucelloses, Pulmonary':ab,ti OR 'Pulmonary Brucelloses':ab,ti OR 'Pulmonary Brucellosis':ab,ti) AND ('Therapeutics'/exp OR Therapeutic OR Therapy OR Therapies OR Treatment OR Treatments:ab,ti)

**CENTRAL(Cochrane Library) (20/09/2022)**

ID Search

#1 MeSH descriptor: [Brucellosis] explode all trees

#2 MeSH descriptor: [Brucella] explode all trees

#3 (Brucelloses):ti,ab,kw OR (Malta Fever):ti,ab,kw OR (Fever, Malta):ti,ab,kw OR (Gibraltar Fever):ti,ab,kw OR (Fever, Gibraltar):ti,ab,kw

#4 (Rock Fever):ti,ab,kw OR (Fever, Rock):ti,ab,kw OR (Cyprus Fever):ti,ab,kw OR (Fever, Cyprus):ti,ab,kw OR (Brucella Infection):ti,ab,kw

#5 (Brucella Infections):ti,ab,kw OR (Infection, Brucella):ti,ab,kw OR (Undulant Fever):ti,ab,kw OR (Fever, Undulant):ti,ab,kw OR (Brucellosis, Pulmonary):ti,ab,kw

#6 (Brucelloses, Pulmonary):ti,ab,kw OR (Pulmonary Brucelloses):ti,ab,kw OR (Pulmonary Brucellosis):ti,ab,kw

#7 #1 OR #2 OR #3 OR #4 OR #5 OR #6

#8 MeSH descriptor: [Therapeutics] explode all trees

#9 (Therapeutic):ti,ab,kw OR (Therapy):ti,ab,kw OR (Therapies):ti,ab,kw OR (Treatment):ti,ab,kw OR (Treatments):ti,ab,kw

#10 #8 OR #9

#11 #7 AND #10
