# Supplementary material for: Better efficacy of triple antibiotics therapy for human brucellosis: A systematic review and meta-analysis
Source: PLoS Negl Trop Dis. 2023 Sep 14;17(9):e0011590. doi: 10.1371/journal.pntd.0011590 (PMC10501551; doi:10.1371/journal.pntd.0011590)
Supplement: S1 Table — (DOCX) [file pntd.0011590.s004.docx]

**S1 Table. Characteristics of included studies.**

| Trial | Patient source | Disease progress | Patient adherence | Basis of sample size calculation | Number of patients(triple/dual) | | |
| --- | --- | --- | --- | --- | --- | --- | --- |
|  |  |  |  |  | Therapeutic failure | Relapse | Adverse reaction |
| Hasanain 2016[1] | The patients were admitted to the departments of Tropical Medicine (Fever Unit) and Internal Medicine | acute/subacute | Five were excluded due to lack of compliance with antimicrobial therapy; Eight more patients were excluded due to inability to attend during the required follow up period | _ | 6/7 | 5/12 | 11/6 |
| Ranjbar 2007[2] | The patients with brucellosis attended the Hamedan Sina Hospital, whether seen as outpatients or as inpatients, were enrolled | _ | Eight were with-drawn: Five patients did not take the prescribed drugs correctly, and three patients had a follow-up period of less than six months | confidence interval of 95% (a=0.05) and study power of 80% (b=0.20); The probability of losing cases was estimated to be 10% (f=10%) | 4/13 | 6/9 | 6/4 |
| Bayindir 2003[3] | The patients with brucellar spondylitis were treated and followed-up at Turgut Ozal Medical Center, Inonu University, Malatya, Turkey | _ | All patients were followed-up for at least 6 months | _ | 0/14 | 0/7 | _ |
| Zhang 2022[4] | The patients with brucellosis diagnosed and treated in Tianjin Second People's Hospital | _ | All patients were followed up within two months | _ | 5/10 | 1/5 | 2/3 |
| Shen 2021[5] | The patients were diagnosed with atypical Brucella osteoarthritis by the Center for Disease Control after undergoing relevant imaging and blood tests at the local hospital | _ | Unclear | _ | 0/4 | _ | 1/3 |
| Sun 2020[6] | The patients with brucellosis were treated in Department of Liver Diseases of Third People’s Hospital of Yingkou City | _ | All patients were followed up within three months | _ | 2/10 | 1/3 | 8/4 |
| Jiang 2020[7] | The patients with brucellosis were treated in Wugang People's Hospital in Hunan Province | _ | All patients were followed up within six months | _ | 3/10 | _ | 5/13 |
| Liang 2018[8] | The patients with brucellosis were treated in Jiyuan People's Hospital in Henan Province | _ | All patients were followed up within two months | _ | 2/10 | 1/7 | 2/3 |
| Sha 2017[9] | Hospitalized patients | acute | All patients were followed up within six weeks | _ | 1/7 | 1/7 | 5/19 |
| Zhou 2016[10] | The patients with brucellosis were treated in Shihezi university school of medicine in the first affiliated hospital of infectious diseases | acute | All patients were followed up within six weeks | _ | 0/0 | 2/10 | 4/3 |
| Yin 2015[11] | The patients with brucellosis were treated in Department of Infection, Xinjiang Bortala Mongol Autonomous Prefecture People's Hospital | acute | All patients were followed up within six months | _ | 0/0 | 0/4 | 9/7 |
| Mile 2012[12] | The patients with brucellosis were treated in University Clinic for Infectious Diseases and Febrile Conditions, Skopje | _ | 57 patients were excluded for the following reasons:  Follow-up time less than 6 months (41 cases), Duration of treatment less than 45 days (9 cases), and treatment-limiting adverse effect (7 cases) | _ | 5/5 | 4/13 | 34/39 |
| Al-Madfaa 2020[13] | The patients who received treatment for blood culture- and/or serology-confirmed brucellosis at King Abdulaziz University Hospital were included | _ | Patients with suspected brucellosis who received no treatment in institution and patients with missing follow up data to determine outcomes were excluded | _ | 5/4 | _ | 7/10 |
| Yang 2021[14] | The patients were admitted to the First Affiliated Hospital of Hebei North University | slow onset | All patients included in the study have completed treatment and follow-up | _ | 8/17 | _ | 9/13 |
| Smailnejad 2012[15] | The patients who were treated in Department of Infectious Diseases at Babol Medical University in Iran | _ | All patients included in the study have completed treatment and one year of follow-up | _ | _ | 1/6 | _ |

**References**

1 Hasanain A, Mahdy R, Mohamed A, Ali M (2016) A randomized, comparative study of dual therapy (doxycycline-rifampin) versus triple therapy (doxycycline-rifampin-levofloxacin) for treating acute/subacute brucellosis Braz J Infect Dis 20(3):250-4. https://doi.org/10.1016/j.bjid.2016.02.004 PMID: 27086734

2 Ranjbar M, Keramat F, Mamani M, Kia AR, Khalilian FO, Hashemi SH, Nojomi M (2007) Comparison between doxycycline-rifampin-amikacin and doxycycline-rifampin regimens in the treatment of brucellosis Int J Infect Dis 11(2):152-6. <https://doi.org/10.1016/j.ijid.2005.11.007> PMID: 16798042

3 Bayindir Y, Sonmez E, Aladag A, Buyukberber N (2003) Comparison of five antimicrobial regimens for the treatment of brucellar spondylitis: a prospective, randomized study J Chemother 15(5):466-71. <https://doi.org/10.1179/joc.2003.15.5.466> PMID: 14598939

4 Cui Z (2022) Effect of Rifampicin Combined with Doxycycline and Levofloxacin in the Treatment of Brucellosis. 35(3). <https://doi.org/10.3969/j.issn.1006-1959.2022.03.027>

5 Licheng S (2021) Analysis of the efficacy of drug therapy alone for patients with atypical Brucella osteoarthritis. China Prac Med 16(31):128-30. <https://doi.org/10.14163/j.cnki.11-5547/r.2021.31.047>

6 Lihui S (2020) Effects of triple therapy of Ofloxacin, Rifampicin and Doxycycline in treatment of patients with brucellosis. Chinese People's Health 32(24):20-1.

7 Li J (2020) Comparison of the efficacy of different antibacterial drug combination regimens in the treatment of brucellosis. Chinese Journal of Clinical Rational Drug Use 13(10):53-4. <https://doi.org/10.15887/j.cnki.13-1389/r.2020.10.030>

8 Chao L, jing L (2018) Effect of different antibacterial drug combination regimens on the treatment effect, recurrence rate and occurrence of adverse effects in brucellosis. Strait Pharmaceutical Journal 30(11):221-2.

9 Rina S (2017) Comparison of different combination therapy regimens of antimicrobial drugs for brucellosis. World Latest Medicine Information 17(58):127-8. <https://doi.org/10.19613/j.cnki.1671-3141.2017.58.067>

10 Yan Z. Efficacy of two antimicrobial regimens in the treatment of brucellosis.: Shihezi University; 2016.

11 Meng Y, Yanhong W, Yu S, Hui L, Lu Z, Minghui L (2015) Comparison of different combination therapy for acute brucellosis. Experimental and Clinical Infectious Diseases 9(06):81-3.

12 Mile B, Valerija K, Krsto G, Ivan V, Ilir D, Nikola L (2012) Doxycycline-rifampin versus doxycycline-rifampin-gentamicin in treatment of human brucellosis Trop Doct 42(1):13-7. <https://doi.org/10.1258/td.2011.110284> PMID: 22290107

13 Al-Madfaa RO, Alalawi MA, Basudan LO, Alhejaili SF, Eljaaly K, Madani TA, Thabit AK (2020) Dual versus triple therapy for uncomplicated brucellosis: A retrospective cohort study J Infect Dev Ctries 14(12):1380-6. <https://doi.org/10.3855/jidc.12741> PMID: 33378279

14 Yang XM, Jia YL, Zhang Y, Zhang PN, Yao Y, Yin YL, Tian Y (2021) Clinical Effect of Doxycycline Combined with Compound Sulfamethoxazole and Rifampicin in the Treatment of Brucellosis Spondylitis Drug Des Devel Ther 15:4733-40. <https://doi.org/10.2147/dddt.S341242> PMID: 34848945

15 Smailnejad Gangi SM, Hasanjani Roushan MR, Janmohammadi N, Mehraeen R, Soleimani Amiri MJ, Khalilian E (2012) Outcomes of treatment in 50 cases with spinal brucellosis in Babol, Northern Iran J Infect Dev Ctries 6(9):654-9. <https://doi.org/10.3855/jidc.2175> PMID: 23000864
