# Supplementary material for: Better efficacy of triple antibiotics therapy for human brucellosis: A systematic review and meta-analysis
Source: PLoS Negl Trop Dis. 2023 Sep 14;17(9):e0011590. doi: 10.1371/journal.pntd.0011590 (PMC10501551; doi:10.1371/journal.pntd.0011590)
Supplement: S2 Table — (DOCX) [file pntd.0011590.s005.docx]

**S2 Table. Treatment failure, relapse and adverse reaction rates by regimen and duration of treatment.**

| Trial | Interventions | Relapse | |  | Therapeutic failure | |  | Adverse reaction | |
| --- | --- | --- | --- | --- | --- | --- | --- | --- | --- |
|  |  | event | n(%) |  | event | n(%) |  | event | n(%) |
| Al-Madfaa 2020[1] | D 2×100mg for 45 days+R 4×300mg or 1×900mg for 45 days+S 1000mg for 7 days | - | - |  | 25 | 5(20) |  | 25 | 7(28.0) |
| Bayindir 2003[2] | D 2×100mg for 45 days+R 15mg/kg/day for 45 days+S 1g for 15 days | 22 | 0(0) |  | 22 | 0(0) |  | - | - |
| Hasanain 2016[3], Zhang 2022[4] | D 200mg for 6 weeks+R 900mg for 6 weeks+L 500mg for 6 weeks | 81 | 6(7.4) |  | 92 | 11(12.0) |  | 92 | 13(14.1) |
| Jiang 2020[5], Sun 2020[6], Yin 2015[7] | D 2×100mg for 6 weeks+R 600mg for 6 weeks+L 2×200mg for 7 days | 32 | 0(0) |  | 69 | 3(4.3) |  | 69 | 14(20.3) |
| Liang 2018[8] | D 100mg for 4 weeks+R 2×450mg for 4 weeks+L 400mg for 4 weeks | 36 | 1(2.8) |  | 38 | 2(5.3) |  | 38 | 2(5.3) |
| Mile 2012[9] | D 200mg for 45 days+R 900mg for 45 days+G 2×120mg for 7-10 days | 82 | 4(4.9) |  | 87 | 5(5.7) |  | 87 | 34(39.1) |
| Ranjbar 2007[10] | D 2×100mg for 8 weeks+R 10mg/kg/day for 8 weeks+A 2×7.5mg/kg/day for 7 days | 106 | 6(5.7) |  | 110 | 4(3.6) |  | 110 | 6(5.5) |
| Sha 2017[11] | D 100mg for 6 weeks+R 600mg for 6 weeks+L 400mg for 6 weeks | 55 | 1(1.8) |  | 56 | 1(1.8) |  | 56 | 5(8.9) |
| Shen 2021[12] | D+R+S vs RT (dose is unavailable) | - | - |  | 35 | 0(0) |  | 35 | 1(2.9) |
| Smailnejad 2012[13] | D 2×100mg+R 15mg/kg/day+C 8mg/kg/day | 11 | 1(9.1) |  | - | - |  | - | - |
|  | D 2×100mg+R 15mg/kg/day+G 5mg/kg/day (up to 240mg) | 6 | 0(0) |  | - | - |  | - | - |
|  | D 2×100mg+R 15mg/kg/day+S 1000mg/day | 13 | 0(0) |  | - | - |  | - | - |
| Yang 2021[14] | D 100mg for 4 weeks+R 450mg for 4 weeks+C 100mg for 4 weeks | - | - |  | 50 | 8(16.0) |  | 50 | 9(18.0) |
| Zhou 2016[15], Sun 2020[6] | D 2×100mg for 6 weeks+R 600mg for 6 weeks+L 500mg for 6 weeks | 86 | 3(3.5) |  | 88 | 2(2.3) |  | 88 | 12(13.6) |

D=doxycycline, R=rifampicin, S=streptomycin, T= tetracycline-HCl, L=levofloxacin, C= co-trimoxazole, G= gentamicin

**References**

1 Al-Madfaa RO, Alalawi MA, Basudan LO, Alhejaili SF, Eljaaly K, Madani TA, Thabit AK (2020) Dual versus triple therapy for uncomplicated brucellosis: A retrospective cohort study J Infect Dev Ctries 14(12):1380-6. <https://doi.org/10.3855/jidc.12741> PMID: 33378279

2 Bayindir Y, Sonmez E, Aladag A, Buyukberber N (2003) Comparison of five antimicrobial regimens for the treatment of brucellar spondylitis: a prospective, randomized study J Chemother 15(5):466-71. <https://doi.org/10.1179/joc.2003.15.5.466> PMID: 14598939

3 Hasanain A, Mahdy R, Mohamed A, Ali M (2016) A randomized, comparative study of dual therapy (doxycycline-rifampin) versus triple therapy (doxycycline-rifampin-levofloxacin) for treating acute/subacute brucellosis Braz J Infect Dis 20(3):250-4. <https://doi.org/10.1016/j.bjid.2016.02.004> PMID: 27086734

4 Cui Z (2022) Effect of Rifampicin Combined with Doxycycline and Levofloxacin in the Treatment of Brucellosis. 35(3). <https://doi.org/10.3969/j.issn.1006-1959.2022.03.027>

5 Li J (2020) Comparison of the efficacy of different antibacterial drug combination regimens in the treatment of brucellosis. Chinese Journal of Clinical Rational Drug Use 13(10):53-4. <https://doi.org/10.15887/j.cnki.13-1389/r.2020.10.030>

6 Lihui S (2020) Effects of triple therapy of Ofloxacin, Rifampicin and Doxycycline in treatment of patients with brucellosis. Chinese People's Health 32(24):20-1.

7 Meng Y, Yanhong W, Yu S, Hui L, Lu Z, Minghui L (2015) Comparison of different combination therapy for acute brucellosis. Experimental and Clinical Infectious Diseases 9(06):81-3.

8 Chao L, jing L (2018) Effect of different antibacterial drug combination regimens on the treatment effect, recurrence rate and occurrence of adverse effects in brucellosis. Strait Pharmaceutical Journal 30(11):221-2.

9 Mile B, Valerija K, Krsto G, Ivan V, Ilir D, Nikola L (2012) Doxycycline-rifampin versus doxycycline-rifampin-gentamicin in treatment of human brucellosis Trop Doct 42(1):13-7. <https://doi.org/10.1258/td.2011.110284> PMID: 22290107

10 Ranjbar M, Keramat F, Mamani M, Kia AR, Khalilian FO, Hashemi SH, Nojomi M (2007) Comparison between doxycycline-rifampin-amikacin and doxycycline-rifampin regimens in the treatment of brucellosis Int J Infect Dis 11(2):152-6. <https://doi.org/10.1016/j.ijid.2005.11.007> PMID: 16798042

11 Rina S (2017) Comparison of different combination therapy regimens of antimicrobial drugs for brucellosis. World Latest Medicine Information 17(58):127-8. <https://doi.org/10.19613/j.cnki.1671-3141.2017.58.067>

12 Licheng S (2021) Analysis of the efficacy of drug therapy alone for patients with atypical Brucella osteoarthritis. China Prac Med 16(31):128-30. <https://doi.org/10.14163/j.cnki.11-5547/r.2021.31.047>

13 Smailnejad Gangi SM, Hasanjani Roushan MR, Janmohammadi N, Mehraeen R, Soleimani Amiri MJ, Khalilian E (2012) Outcomes of treatment in 50 cases with spinal brucellosis in Babol, Northern Iran J Infect Dev Ctries 6(9):654-9. <https://doi.org/10.3855/jidc.2175> PMID: 23000864

14 Yang XM, Jia YL, Zhang Y, Zhang PN, Yao Y, Yin YL, Tian Y (2021) Clinical Effect of Doxycycline Combined with Compound Sulfamethoxazole and Rifampicin in the Treatment of Brucellosis Spondylitis Drug Des Devel Ther 15:4733-40. <https://doi.org/10.2147/dddt.S341242> PMID: 34848945

15 Yan Z. Efficacy of two antimicrobial regimens in the treatment of brucellosis.: Shihezi University; 2016.
