# Supplementary material for: Better efficacy of triple antibiotics therapy for human brucellosis: A systematic review and meta-analysis
Source: PLoS Negl Trop Dis. 2023 Sep 14;17(9):e0011590. doi: 10.1371/journal.pntd.0011590 (PMC10501551; doi:10.1371/journal.pntd.0011590)
Supplement: S2 Fig — (DOCX) [file pntd.0011590.s007.docx]

| (a) | 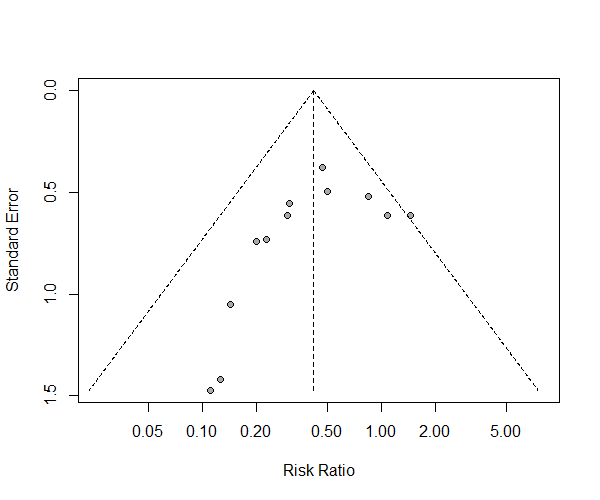 |  | (b) | 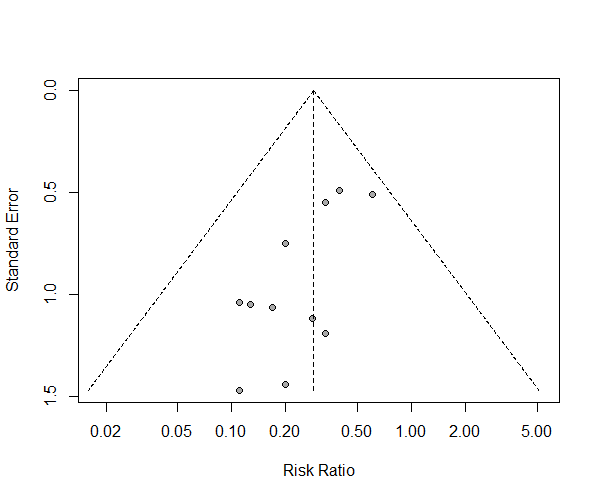 |
| --- | --- | --- | --- | --- |
| (c) | 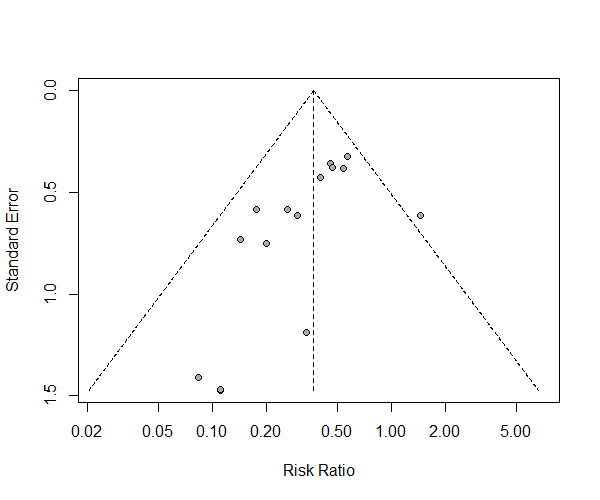 |  | (d) | 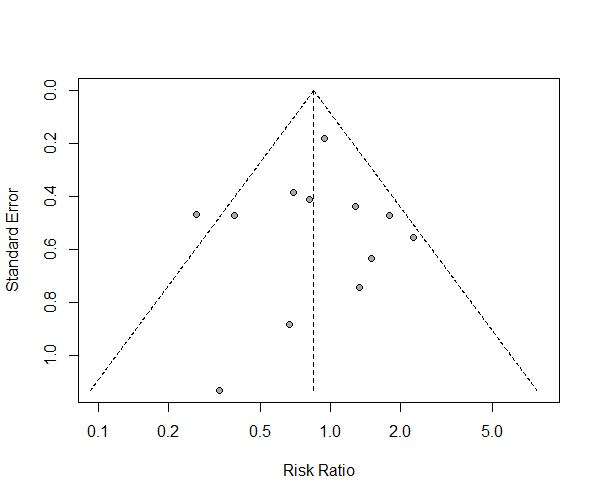 |

**S2 Fig. Funnel plot of overall outcome indicators. (a) Therapeutic failure rate. (b) Relapse rate. (c) Overall therapeutic failure rate. (d) Side effect rate.**
